# Description of a new species of the genus *Riukiaria* (Diplopoda, Polydesmida, Xystodesmidae) from eastern China, with the characterization of its complete mitochondrial genome

**DOI:** 10.3897/zookeys.1272.182977

**Published:** 2026-03-10

**Authors:** Zewu Han, Xin Liu, Jiajia Wang, Yan Dong

**Affiliations:** 1 College of Biology and Food Engineering, Chuzhou University, Chuzhou 239000, China College of Biology and Food Engineering, Chuzhou University Chuzhou China; 2 School of Life Sciences and Medical Engineering, Anhui University, Hefei 230601, China School of Life Sciences and Medical Engineering, Anhui University Hefei China

**Keywords:** Complete mitochondrial genome, identification key, millipede, mitogenome, *
Riukiaria
*, species description, taxonomy

## Abstract

This study describes a new millipede species of the genus *Riukiaria* from China: *Riukiaria langyaensis***sp. nov**., and provides an identification key to all 19 *Riukiaria* species currently known from China. A systematic review was conducted on the 34 described *Riukiaria* species, and these species were classified into five categories based on the morphology of the male gonopod. The complete mitochondrial genome of this new species was sequenced and annotated for the first time, representing the first mitogenomic data reported for the genus *Riukiaria*. The mitogenome of *R. langyaensis***sp. nov**. is 14,954 bp in length, containing 13 protein-coding genes, 22 tRNA genes, 2 rRNA genes, and a control region, with an overall A+T content of 65.8%. All 37 mitochondrial genes are encoded on the Heavy strand. The results help clarify the confused species relationships within *Riukiaria* and provide fundamental data for phylogenetic studies of Xystodesmidae.

## Introduction

The genus *Riukiaria* was first proposed by Verhoeff (1936), but it was invalid because no type species was designated. The genus *Riukiaria* had not been validly established until Attems (1938) designated *R. pugionifera* Verhoeff, 1936 as the type species, making Attems its valid author. *Riukiaria* is one of the most species-rich genera in the warm-temperate to subtropical regions of East Asia, with approximately 38 species currently described (Marek et al. 2014; Golovatch 2015; Nguyen 2016; Chen et al. 2023; Korsós and Nakamura 2025). Species of the genus *Riukiaria* are primarily distributed in southern China and southern Japan (Marek et al. 2014). Only one species, *R. cucfuongensis* Nguyen, 2016, has been recorded from Vietnam.

Due to their morphological similarities, many *Riukiaria* species were originally assigned to other genera such as *Rhysodesmus* and *Rhysolus* (Gressitt 1941; Takakuwa 1941, 1942; Miyosi 1952a, 1952b, 1957; Wang 1956, 1957; Haga 1968; Golovatch 1978). Shinohara (1977) later clarified the taxonomy of this group, indicating that all East Asian species previously placed in *Rhysodesmus* should be transferred to one of two genera: *Riukiaria* Attems (a synonym of which is *Rhysolus* Chamberlin & Wang, 1953), or *Takakuwaia* Verhoeff, 1936—which Hoffman (1956) regarded as a junior synonym of *Xystodesmus* Cook, 1895. Accordingly, *Rhysodesmus* Cook, 1895 has been appropriately restricted to include only North American species (Hoffman 1980).

The genera *Riukiaria* and *Xystodesmus* Cook, 1895 are placed in the tribe Xystodesmini as sister groups (Golovatch 2014). The primary criterion for distinguishing between species of these two genera lies in gonopod morphology: unlike the simple, bifurcated, forceps-like gonopods of most *Riukiaria* species, those of *Xystodesmus* are usually slightly more complex, with additional branches and appendages (Korsós and Nakamura 2025).

To date, only a limited number of studies have attempted to elucidate the phylogenetic relationships of *Riukiaria* and *Xystodesmus* using molecular genetic data (Sota and Tanabe 2010; Nakamura and Korsós 2011; Means et al. 2021). Based on mitochondrial *COX1–COX2* sequences (Sota and Tanabe 2010), mitochondrial rRNA genes (Nakamura and Korsós 2011), and ultimately combined mitochondrial and nuclear gene fragments (Means et al. 2021), a series of phylogenetic analyses has consistently identified *Riukiaria* and *Xystodesmus* as sister genera. Historically, complete mitochondrial genomes have been widely employed in phylogeographic and phylogenetic studies due to their rapid evolutionary rate, simple structure, and high information content (Castellana et al. 2011; Iwasaki et al. 2013; Cameron 2014). Approximately 400 species in the family Xystodesmidae have been documented morphologically, yet only two species—*Appalachioria falcifera* and *Xystodesmus* sp. YD-2016—have mitogenomic sequence data available in the National Center for Biotechnology Information (NCBI) (Brewer et al. 2013; Dong et al. 2016).

To improve species identification and advance understanding of the phylogenetic relationships within *Riukiaria* and with other millipede genera, the present study describes the morphological characteristics of *Riukiaria langyaensis* sp. nov. and reports its complete mitochondrial genome sequence.

## Material and methods

### Morphological study

The specimens were collected with tweezers from Langya Mountain Forest Farm (32°25'60"N, 118°28'09"E) and Niutou Mountain (32°27'47"N, 118°29'70"E), Chuzhou, Anhui Province, China. Live animals were first observed and photographed with a Canon EOS 5D Mark VI camera with a Canon EF 100 mm macro lens. For UV illumination, an AUX Black Light Blue (10 W) lamp was used. Most specimens were preserved in 75% ethanol for morphological studies, and the remainder were preserved in absolute ethanol and stored at -80 °C for molecular research. All specimens are deposited in the Natural Museum of Chuzhou University, Chuzhou, Anhui, China.

Specimens were examined, photographed and measured using a ZEISS SteREO Discovery.V8 stereomicroscope (Germany) equipped with an Axiocam 208 color camera. The sex of specimens was determined by the presence or absence of gonopods on the ventral side of the seventh body ring.

### Mitochondrial genome sequencing, annotation and analyses

Based on a single male individual, a total of 60 Gb of high-quality genomic data were generated using the Paired-end 150 bp sequencing strategy on the Illumina Nova Seq 6000 platform by Berry (Beijing, China). The mitochondrial genome was assembled and annotated using GENEIOUS PRIME v. 2023.2.1 (https://www.geneious.com), the MITOS WEB SERVER (http://mitos.bioinf.uni-leipzig.de/index.py), the TRNASCAN-SE SEARCH SERVER, ARWEN software, ORF FINDER (https://www.ncbi.nlm.nih.gov/orffinder/) and OGDRAW (https://chlorobox.mpimp-golm.mpg.de/OGDraw) (Laslett and Canbäck 2008; Bernt et al. 2013; Lowe and Chan 2016; Greiner et al. 2019). Nucleotide frequencies and the relative synonymous codon usage (RSCU) were calculated using MEGA 11 software (Tamura et al. 2021). After publication, the mitogenome sequences will be available in the NCBI database (https://www.ncbi.nlm.nih.gov/) under the accession number PX436095.

## Taxonomic

### Class Diplopoda de Blainville in Gervais, 1844

**Subclass Chilognatha Latreille, 1802/1803**

**Order Polydesmida Pocock, 1887**

**Superfamily Xystodesmoidea Cook, 1895**

**Family Xystodesmidae Cook, 1895**

#### Genus Riukiaria

Taxon classificationAnimaliaPolydesmidaXystodesmidae

Attems, 1938

12E40BB3-BBB4-5C95-88F5-E67A7763F921

##### Type species.

*Riukiaria pugionifera* Verhoeff, 1936.

##### Generic diagnosis.

Body with 20 rings, compact and relatively small, displaying an often vivid colour pattern. Posterolateral corners of paraterga blunt on rings 1–4, becoming acutely produced on rings 5–18. Gonopods simple, biramous, and forceps-like; solenomere consistently longer and stouter than prefemoral process (when present). Gonopod coxite with 0–2 setae (Korsós et al. 2011; Golovatch 2014).

##### Included species or subspecies.

*Riukiaria anachoreta* Tanabe, 1988, *R. belousovi* Golovatch, 2014, *R. bifida* (Takakuwa, 1942), *R. capaca* Wang & Zhang, 1993, *R. chelifera* (Takakuwa, 1941), *R. chinensis* Tanabe, Ishii & Yin, 1996, *R. cohaesiva* (Wang, 1957), *R. contigua* (Wang, 1957), *R. cornuta* (Haga, 1968), *R. cucfuongensis* (Nguyen, 2016), *R. datei* (Miyosi, 1957), *R. davidiani* Golovatch, 2014, *R. diacantha* (Miyosi, 1952), *R. falcifera* Verhoeff, *R. geniculata* (Takakuwa, 1941), *R. holstii* (Pocock, 1895), *R. jamila* Tanabe, 1990, *R. kabaki* Golovatch, 2014, *R. korolevi* Golovatch, 2014, *R. maculata* Korsós, Nakamura & Tanabe, 2011, *R. marinae* (Golovatch, 1978), *R. martensi* Golovatch, 2014, *R. montana* (Haga, 1968), *R. mundyi* Korsós, Nakamura & Tanabe, 2011, *R. ochracea* (Gressitt, 1941), *R. puella* Tanabe, 1988, *R. pugionifera* Verhoeff, 1936, *R. rosulans* (Tömösváry, 1885), *R. scutata* (Takakuwa, 1942), *R. semicircularis semicircularis* (Takakuwa, 1941), *R. semicircularis hosidei* (Miyosi, 1952), *R. spatuliformis* Golovatch, 2015, *R. spina* Chen, Zheng & Jiang, 2023, *R. spiralipes* (Takakuwa, 1942), *R. taiwana* (Takakuwa, 1942), *R. tianmu* (Tanabe, Ishii & Yin, 1996), *R. uncata* (Haga, 1968), *R. uraensis* (Wang, 1956).

#### Riukiaria langyaensis

Taxon classificationAnimaliaPolydesmidaXystodesmidae

Han & Wang sp. nov.

4DC1038E-EAA9-5AD4-A30A-68F74816DFE8

https://zoobank.org/3CBBEFF0-1451-48E1-A199-79D528B02B65

Figs 1, 2, 3, 4, 5

##### Material examined.

***Holotype***: China • ♂, Anhui, Chuzhou City, Langya Mountain Forest Farm, 32°25'59"N, 118°28'09"E, alt. 100 m, 7 May 2025, Z.W. Han & J.J Wang leg, NMCHZU DH28. GenBank: PX436095. ***Paratypes***: • 1♀, same locality and date as holotype, NMCHZU DH29.

**Figure 1.**
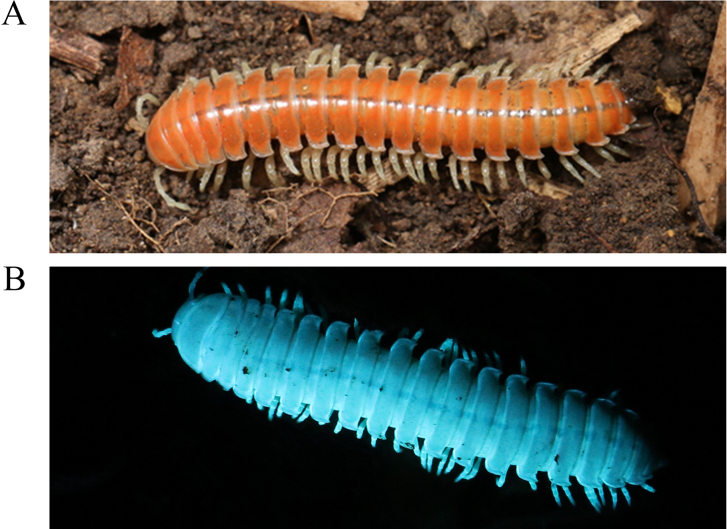
*Riukiaria langyaensis* sp. nov. **A**. Live coloration; **B**. Live specimen under UV light.

**Figure 2.**
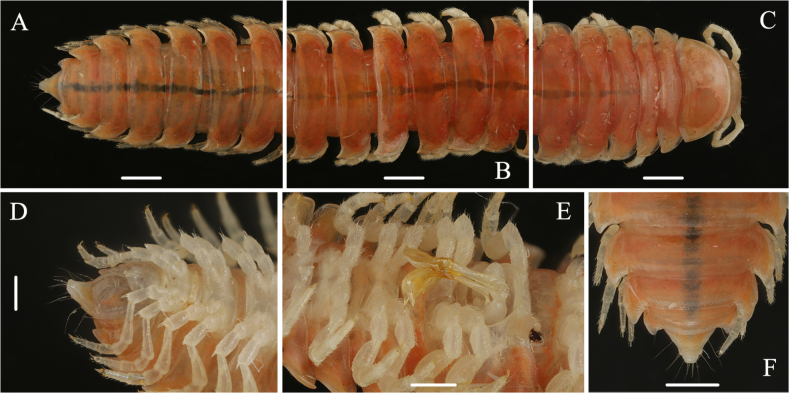
*Riukiaria langyaensis* sp. nov. **A**. Posterior part of body, dorsal view; **B**. Midbody rings, dorsal view; **C**. Anterior part of body, dorsal view; **D**. Telson, ventral view; **E**. Both gonopods, ventral view; **F**. Rings 16–19 and telson, dorsal view. Scale bars: 1.0 mm.

**Figure 3.**
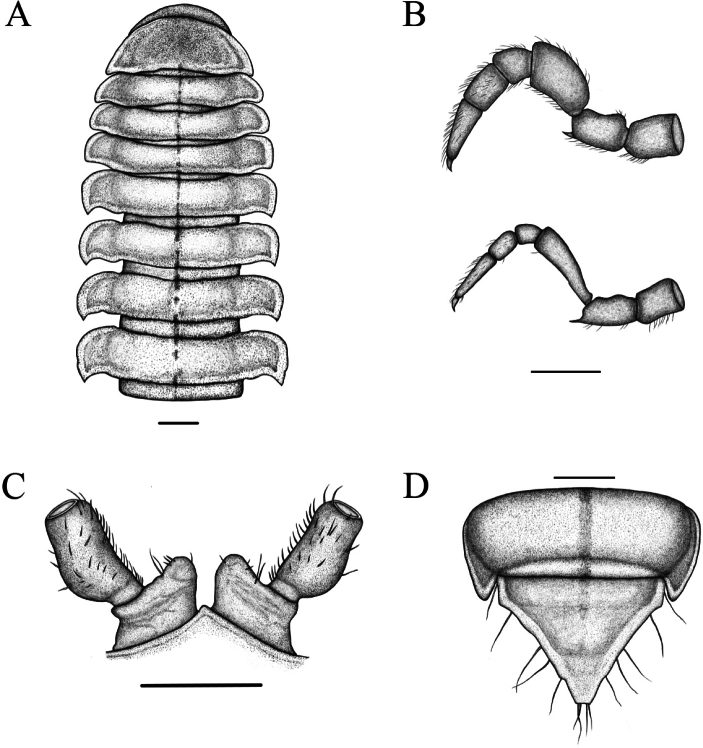
*Riukiaria langyaensis* sp. nov. **A**. Anterior body part, dorsal view; **B**. 8^th^ and 16^th^ pairs of legs, left legs, anterior view; **C**. Sternum, coxa, and prefemur of 2^nd^ leg pair, posterior view; **D**. Epiproct and 19^th^ ring, dorsal view. Scale bars: 1.0 mm.

**Figure 4.**
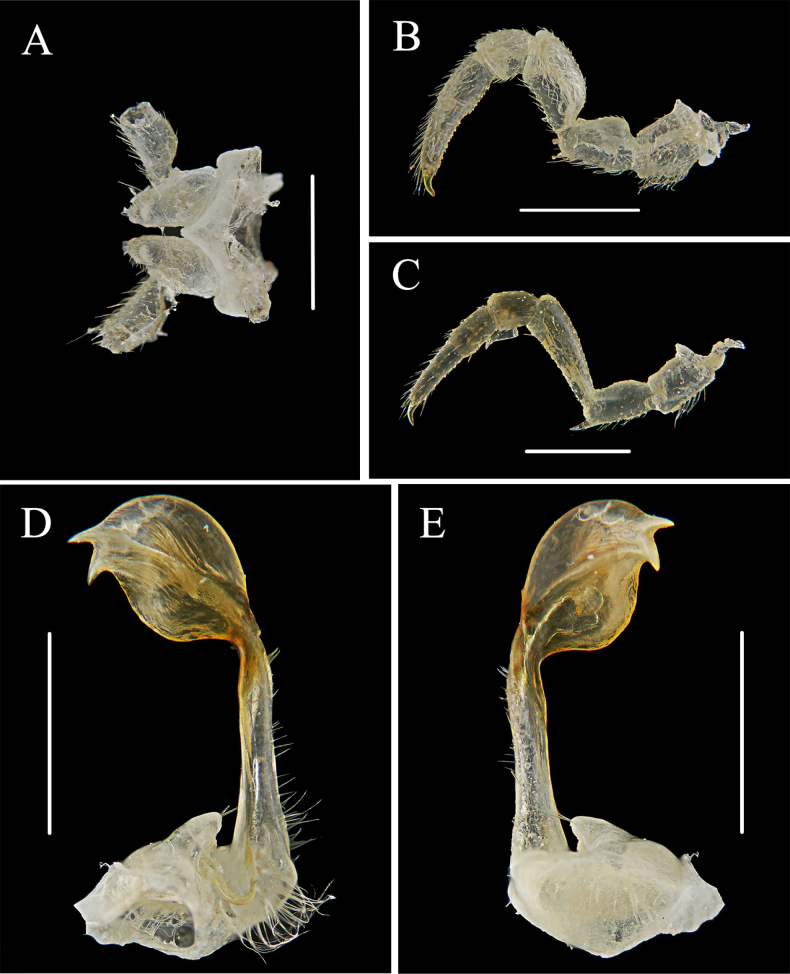
*Riukiaria langyaensis* sp. nov. **A**. Sternum, coxa, and prefemur of 2^nd^ leg pair, posterior view; **B**. 8^th^ pair of legs, left leg, anterior view; **C**. 16^th^ pair of legs, left leg, anterior view; **D**. Left gonopod, mesal view; **E**. Left gonopod, lateral view. Scale bars: 1.0 mm.

**Figure 5.**
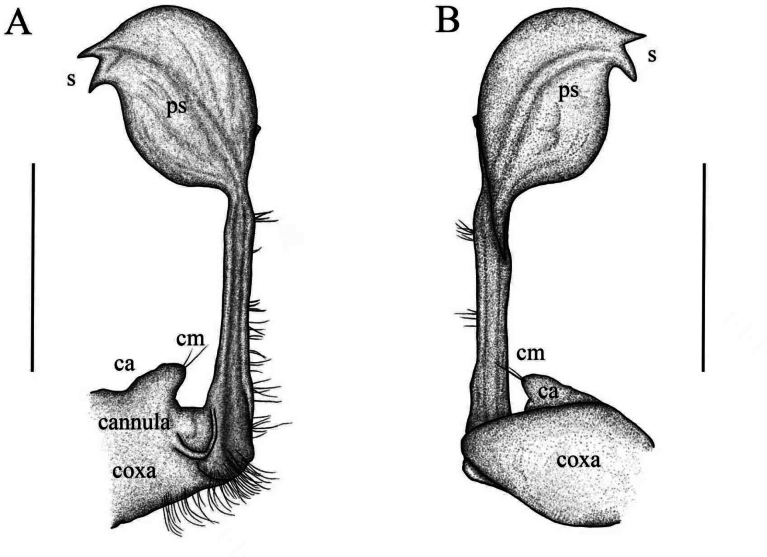
*Riukiaria langyaensis* sp. nov. **A**. Left gonopod, mesal view; **B**. Left gonopod, lateral view. Abbreviations: cm = coxal macroseta, ca = coxal apophysis, ps = prostatic groove, s = solenomere. Scale bars: 1.0 mm.

##### Other material.

China • 3♂2♀, Anhui, Chuzhou County, Niutou Mountain, 32°27'47"N, 118°29'97"E, alt. 87 m, 25 May 2025, Z.W. Han & J.J Wang leg, NMCHZU DH30-34.

##### Diagnosis.

This species closely resembles *Riukiaria mundyi* Korsós, Nakamura & Tanabe, 2011 in tergal features and body coloration. However, *Riukiaria langyaensis* sp. nov. differs by having a lighter body color than *R. mundyi*, with antennae and walking legs exhibiting a wheat-colored hue and the paratergal margins appearing translucent. In terms of gonopod structure, the prefemorite of *Riukiaria langyaensis* sp. nov. is similar to that of *Riukiaria contigua* (Wang, 1957), but its acropodite is more inflated and nearly spherical, with an indistinctly forked tip, and the prefemoral processes are completely missing.

##### Description.

Length of holotype 23.6 mm, midbody paranotal width 4.8 mm, metatergal width 2.9 mm. Female body length 25.1 mm, midbody paranotal width 5.1 mm, metatergal width 3.4 mm.

Coloration in life nearly uniform dark orange, with posterior ^2^/_5_ of body occasionally pale orange in some individuals. Epicranium concolorous with body; antennae and legs wheat-coloured. No sexual dimorphism observed. Under UV light, entire body exhibiting strong sky-blue fluorescence. In alcohol, vivid coloration fading rapidly, most metaterga becoming light marbled orange.

Body with 20 rings. Pro- and metazonae smooth, devoid of tubercles, punctation or wrinkles, except for corrugated folds on ventral side of metaterga. Head smooth, epicranial suture distinct; forehead raised, surface uneven. Mandibular stipes ventrally depressed. Several setae scattered above clypeus, with two dense transverse rows at its margin and on labrum. Antennae straight, reaching behind midway of body ring 3 when stretched dorsally; first antennomere globose, second slightly clavate, subequal with antennomeres 3–5, sixth longest, c. 1.2 times as long as the fifth, seventh small, slender, slightly longer than wide.

In width, head < collum < ring 2 < 3 < 4 = 11, thereafter body gradually tapering towards telson. Collum convex, smooth, shiny; lateral and posterior margin with weak ridge, lateral corners triangular, slightly directed caudad. All paraterga clearly delimited by a dorsal sulcus, lateral calluses broad and distinct; margins translucent. Caudolateral corner of paraterga invariably rounded, in collum acute, in rings 2–4 subrectangular, in following rings growing increasingly acute and drawn well caudad behind rear tergal margin, nearly beak-shaped in posterior rings (Fig. 2A–C). Pore formula normal (5, 7, 9, 10, 12, 13, 15–18), ozopores lying in median excavation of paraterga (in lateral view). Epiproct short, in dorsal view subtriangular, in lateral view protruding, parallel-sided, slightly curved ventrad, with 7+7 setae, 2+2 of them on knobs. Paraprocts strongly marginate with 2+2 setae; hypoproct with 1+1 setae on knobs (Fig. 2F).

Sterna without modifications, mostly smooth; very narrow between legs 1 and 2, clearly broader until ring 7, especially broad behind gonopods. Legs generally densely setose, robust, c. 1.1–1.2 times as long as midbody height, last pair considerably shorter than previous ones (Fig. 4B, C). Coxae 3–7 especially densely setose ventrally. Prefemora with a moderately developed ventral spine, growing increasingly stronger towards body end; femur straight, c. 2.2 times as long as prefemur; postfemur stout; tibia straight, subequal to postfemur; tarsus slender, c. 1.8 times as long as tibia; claw curved, c. 0.25 mm long. Coxa 2 with a strong, median projection about half as long as coxa proper apically with a membranous tubula surrounded by strong setae; prefemoral joint with 1+1 macrosetae (Fig. 4A).

Gonopod aperture transversely oblong, c. 1.75 times as broad as long, with elevated lateral and caudal edges. Gonopod coxa short, stout, subcylindrical, with two coxal macrosetae, and a normal, prominent, mesal cannula. Prefemoral process entirely absent. Prefemorite with dense setae on left side. Acropodite inflated, oval, distinctly curved, with a prostatic groove. Solenomere apical, bifurcated (Fig. 4D, E).

##### Distribution.

Known only from the type locality in Chuzhou, Anhui Province, China.

##### Etymology.

The species name is derived from the geographic name of the location where the species was first discovered.

##### Remarks.

The genus *Riukiaria* comprises 38 species distributed across China, Japan, and Vietnam (Marek et al. 2014; Golovatch 2015; Chen et al. 2023; Korsós and Nakamura 2025). In China, 18 species have been reported from Shaanxi (1 species), Sichuan (5 species), Zhejiang (2 species), Fujian (1 species), Chongqing (1 species), and Taiwan (8 species). *Riukiaria langyaensis* sp. nov. is the first species of *Riukiaria* reported from Anhui Province.

### Mitogenome structure and organization

**Figure 6.**
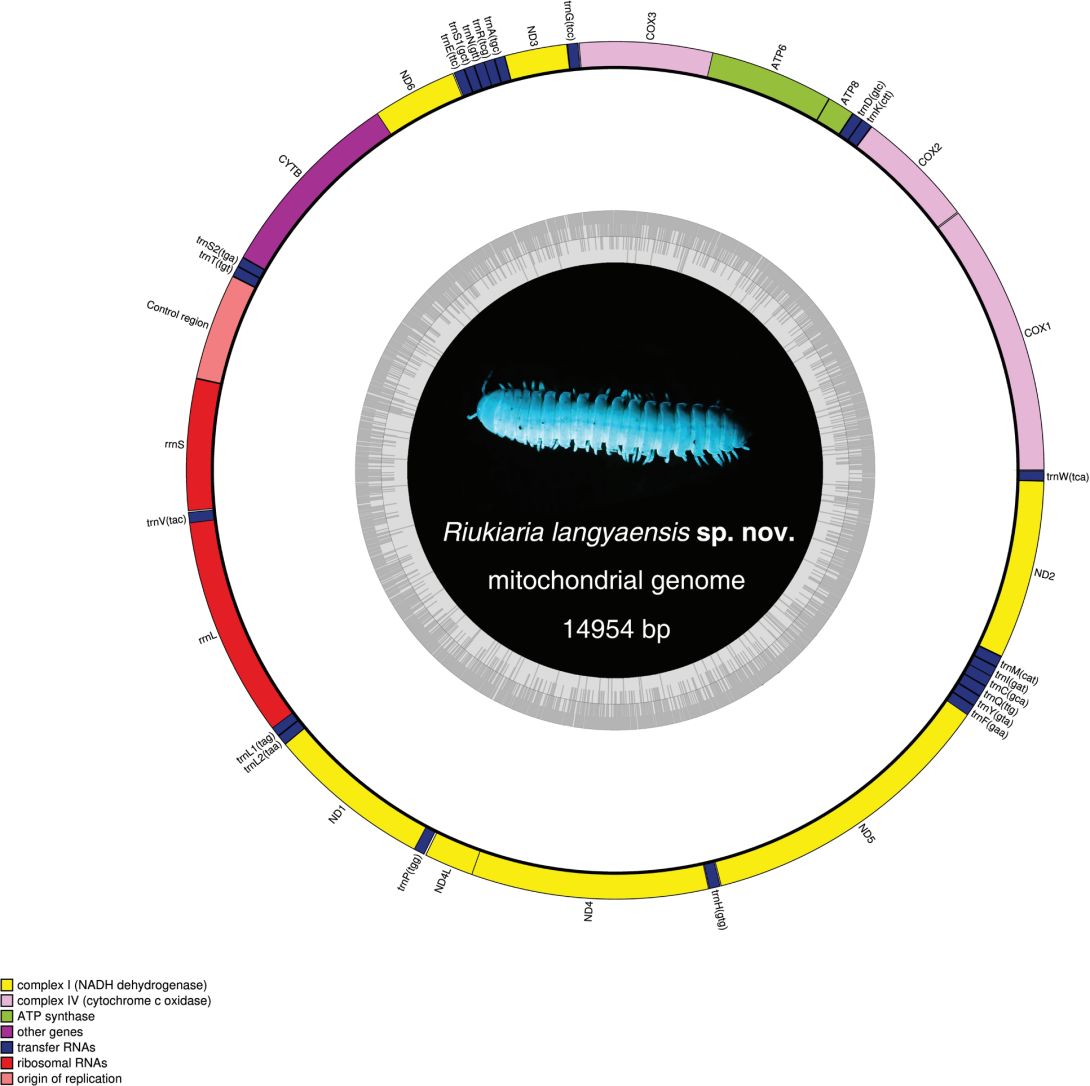
Circular representation of the mitogenome of *Riukiaria langyaensis* sp. nov. The photo in the center shows a live specimen under UV light.

The complete mitochondrial genome of *Riukiaria langyaensis* sp. nov. is a typical double-stranded circular DNA molecule of 14,954 bp in length (Fig. 6). It encodes 37 typical mitochondrial genes, including 13 protein-coding genes (PCGs), 22 tRNA genes, two rRNA genes, and a control region (CR), with an overall A+T content of 65.8%. This composition is consistent with the general structure of metazoan mitochondrial DNA but exhibits several unique characteristics. All 37 mitochondrial genes are encoded on the Heavy strand. A total of 11 gene overlaps were identified, with the longest (40 bp) occurring between *rrnL* and *trnL1*. In contrast, eight intergenic spacer regions were detected, the longest of which (7 bp) lies between *trnP* and *ND4L*. Relevant mitochondrial genome structure information is provided in Suppl. material 1: table S1.

The 13 PCGs in the *Riukiaria langyaensis* mitogenome have a combined length of 11,060 bp, accounting for 73.96% of the genome. All PCGs are initiated by standard start codons (ATG, ATT, or ATA), except for *ND2*, which starts with GTG. The occurrence of non-standard start codons has also been documented in other millipedes, such as *ND3* and *ND4* in *Xystodesmus* sp. and *Asiomorpha coarctata* (Dong et al. 2016). Most PCGs terminate with the canonical stop codons TAA or TAG, whereas *COX2*, *ND1*, *ND5*, and *CYTB* use incomplete stop codons (represented only by T or TA). Such abbreviated stop codons are commonly observed in other arthropods and are presumed to be post-transcriptionally modified to TAA or TAG to enable proper translation termination. The PCGs encode a total of 3676 amino acids. Codons ending with A or U are significantly more frequent than those ending with C or G, reflecting a strong AT bias at the third codon position—a pattern consistent with previous findings in Myriapoda. The relative synonymous codon usage (RSCU) values and the codon usage frequency figure are provided in Suppl. material 1: fig. S1.

**Figure 7.**
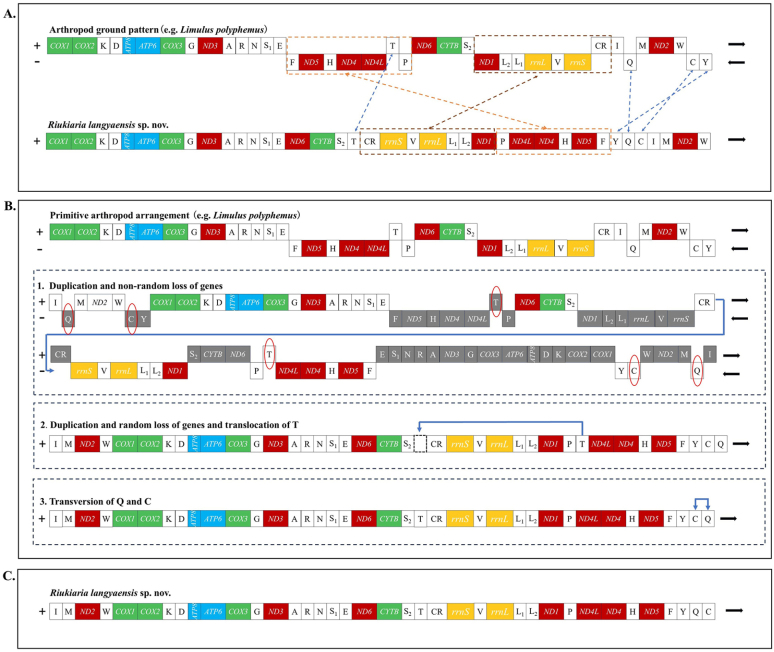
Inferred intermediate steps between the ancestral gene arrangement of myriapod and *Riukiaria langyaensis* sp. nov. mitogenomes. PCGs, CR, and tRNAs are indicated with boxes. Genes labeled above the diagram are encoded on the Heavy strand, and those below the diagram on the Light strand. The lost genes are labeled in gray. **A**. Ancestral gene arrangement of myriapod; **B**.(1) Two monomers derived from the duplication of an ancestor, arranged in a circular dimer. Subsequently, non-random loss is followed according to the orientation of transcription for each gene; **B**.(2) Tandem duplication followed by random deletion (TDRL) leads to the translocation of *trnT*; **B**.(3) Recombination model leads to transversion of *trnC*-*trnQ*; **C**. Final result of the genetic rearrangement of the *Riukiaria langyaensis* sp. nov. mitogenome.

Compared with ancestral arthropods (e.g., *Limulus polyphemus*), the mitochondrial genome of *Riukiaria langyaensis* sp. nov. exhibits rearrangements involving seven genes and gene blocks (including *trnF*-*nad5*-*trnH*-*nad4*-*nad4L*-*trnT*-*trnP*, *ND1*-*trnL2*-*trnL1*-*rrnL*-*trnV*-*rrnS*, *trnT*, *trnC*, *trnY*, *trnI*, and *trnQ*), as well as the AT-rich region (the control region, CR). The mitogenome of *R. langyaensis* sp. nov. is unique among myriapod species, with all coding regions located on a single strand. Currently, several well-established mechanisms, including duplication-random loss (TDRL) (Moritz and Brown 1987), duplication-nonrandom loss (TDNL) (Lavrov et al. 2002), and homologous recombination (Lunt and Hyman 1997), are commonly used to explain mitochondrial gene rearrangements in animals. However, the rearrangement pattern of *R. langyaensis* sp. nov. displays several distinctive features that cannot be directly explained by these existing models. Based on the mechanism of “genome-scale duplication + (non-random/random) loss + recombination”, Zuo et al. (2022) proposed a new rearrangement model termed “TD(N/R)L + RC”. This model is also applicable to interpreting the gene rearrangement process in this species: First, the entire mitochondrial genome underwent tandem duplication, resulting in a dimeric molecule composed of two identical monomers linked head-to-tail. This was followed by non-random loss of duplicated genes across consecutive copies. Subsequently, *trnT* was translocated, and *trnC* and *trnQ* were inverted. Notably, the 3' end of monomer 1 is linked to the 3' end of monomer 2, ultimately forming the current gene arrangement of the *R. langyaensis* sp. nov. mitochondrial genome (Fig. 7).

## Discussion

Following the formal synonymization of *Koreoaria* Verhoeff, 1937 with *Xystodesmus* by Korsós and Nakamura (2025), the genera that represent the tribe Xystodesmini in East Asia have been reduced from five to four, now comprising: *Levizonus* Attems, 1938; *Riukiaria* Attems, 1938; *Xystodesmus* Cook, 1895; and *Yaetakaria* Hoffman, 1949. Both morphological and molecular phylogenetic studies indicate that *Riukiaria* and *Xystodesmus* are most closely related. They exhibit striking similarities in body size, coloration, and gonopod structure. Species discrimination between these two genera primarily relies on male gonopod structure: *Riukiaria* typically exhibits simple, forked, forceps-shaped gonopods, whereas those of *Xystodesmus*, while also forceps-shaped, are structurally more complex and often possess additional branches or appendages. However, significant intrageneric variation exists within these closely related genera, making it challenging to assign preserved specimens to the correct genus based solely on morphological characteristics.

In molecular phylogenetics, the use of mitochondrial *COX1* gene sequences alone for tree reconstruction presents inherent limitations, typically characterized by low topological resolution and weak support at key nodes. The phylogenetic tree we constructed within the tribe Xystodesmini using partial *COX1* gene sequences exemplifies these shortcomings, particularly in the unresolved boundaries and interspecific intermixing among closely related genera such as *Riukiaria*, *Xystodesmus*, and *Levizonus* (see Suppl. material 2 for detailed methods, results, and phylogenetic trees). This outcome can be attributed to two main factors. First, although *COX1* is widely used in DNA barcoding and exhibits high variability among closely related species, its limited number of informative sites often leads to signal saturation or insufficient phylogenetic signal when resolving deeper branches at the generic level and above. Second, single-gene trees are susceptible to evolutionary processes such as incomplete lineage sorting, gene flow, and hybridization, which can obscure the true evolutionary history of species.

Our analysis is further limited by the general scarcity of genomic data for this group. Although approximately 400 species in the family Xystodesmidae have been described morphologically, complete mitochondrial genomes are publicly available for two—*Appalachioria falcifera* and *Xystodesmus* sp. YD-2016. This lack of genomic data constrains high-resolution phylogenetic studies at the genomic scale. Moreover, morphological descriptions for most *Riukiaria* species are outdated, often brief, and lack detailed comparisons or modern illustrations. Together with the limited availability of specimens, these gaps have hindered reliable taxonomic revision and accurate phylogenetic placement within the genus.

**Figure 8.**
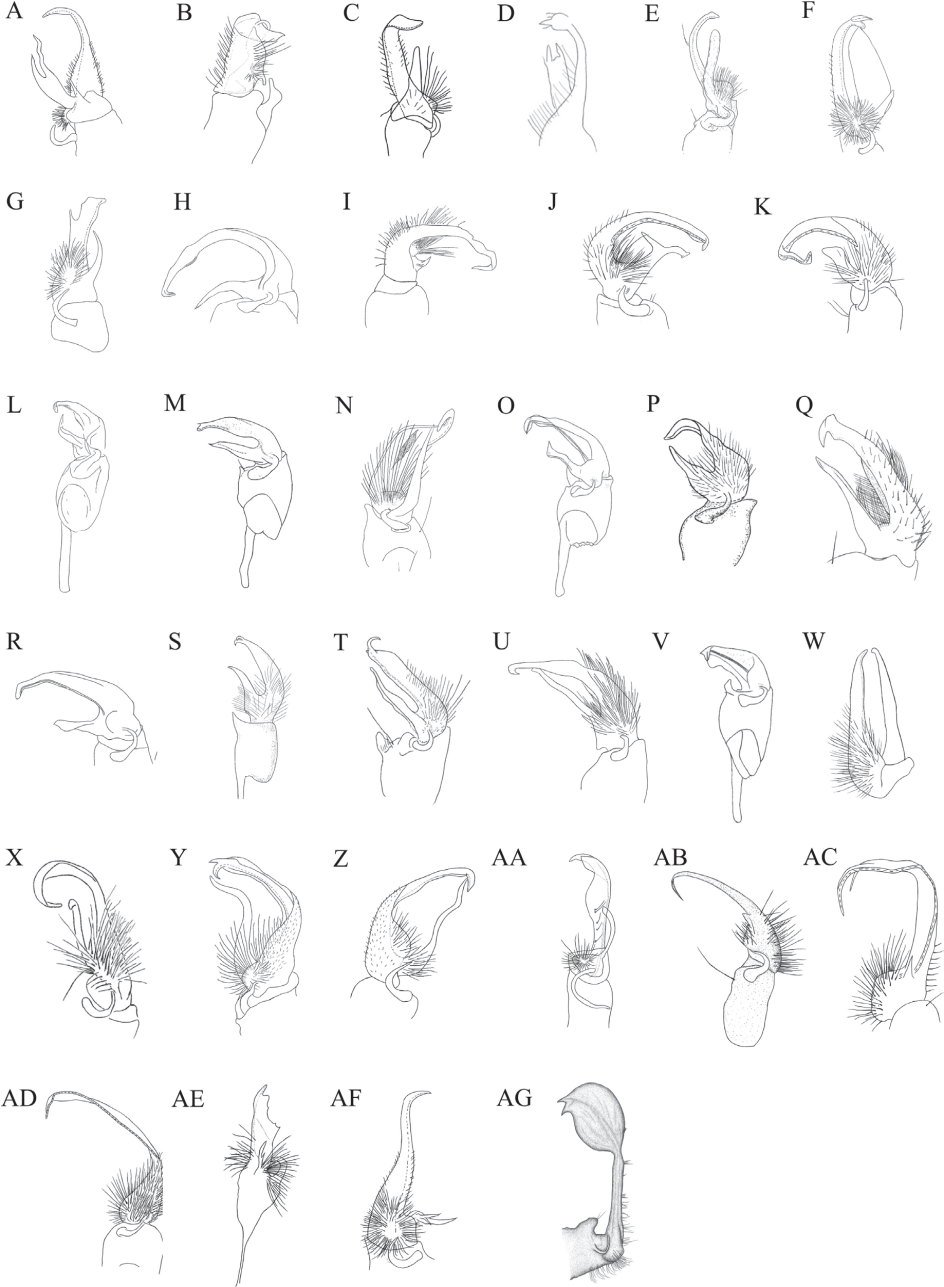
Comparison of gonopods within the genus *Riukiaria*. **A**. *R. bifida*; **B**. *R. cohaesiva*; **C**. *R. geniculata*; **D**. *R. holstii*; **E**. *R. mundyi*; **F**. *R. spiralipes*; **G**. *R. taiwana*; **H**. *R. anachoreta*; **I**. *R. belousovi*; **J**. *R. martensi*; **K**. *R. spatuliformis*; **L**. *R. capaca*; **M**. *R. chinensis*; **N**. *R. datei*; **O**. *R. jamila*; **P**. *R. maculata*; **Q**. *R. marinae*; **R**. *R. puella*; **S**. *R. rosulans*; **T**. *R. semicircularis hosidei*; **U**. *R. spina*; **V**. *R. tianmu*; **W**. *R. chelifera*; **X**. *R. davidiani*; **Y**. *R. diacantha*; **Z**. *R. semicircularis semicircularis*; **AA**. *Riukiaria contigua*; **AB**. *Riukiaria cucfuongensis*; **AC**. *Riukiaria kabaki*; **AD**. *Riukiaria korolevi*; **AE**. *Riukiaria uraensis*; **AF**. *Riukiaria scutata*; **AG**. *Riukiaria langyaensis* sp. nov.

Therefore, an integrative approach combining multi-locus molecular data with detailed morphological re-examination is urgently needed. In this context, we present the first complete mitochondrial genome of *Riukiaria langyaensis* sp. nov., including its sequencing, assembly, and annotation. For the morphological component, we focused on all *Riukiaria* species for which clear gonopod illustrations and detailed morphological descriptions are available, resulting in a total of 34 species included in this analysis. Some early-described species, described solely from brief textual accounts and therefore lacking gonopod illustrations, could not be reliably assigned or compared and were consequently excluded from our study. We redrew the gonopod structures for these 34 species (Fig. 8) and classified them into five types based on male gonopod morphology. The diagnostic features of each gonopod type are outlined below:

Type 1: Pincer-like structure. The prefemur and prefemoral process are distinctly forked, with the prefemur being longer than the prefemoral process. The acropodite is curved at a distinct angle at its tip. The prefemur bears setae; the prefemoral process exhibits a complex morphology in some species (*R. bifida*, *R. cohaesiva*, *R. geniculata*, *R. holstii*, *R. mundyi*, *R. spiralipes* and *R. taiwana*) (Fig. 8A–G).

Type 2: Pincer-like structure. This type is characterized by both the prefemur and the acropodite being bent at a 90° angle (4 species, including *R. anachoreta*, *R. belousovi*, *R. martensi*, and *R. spatuliformis*) (Fig. 8H–K).

Type 3: Pincer-like structure. The prefemur and prefemoral process are compactly arranged. The prefemoral process is slightly shorter than the prefemur and does not form a closed loop with it (11 species, including *R. capaca*, *R. chinensis*, *R. datei*, *R. jamila*, *R. maculata*, *R. marinae*, *R. puella*, *R. rosulans*, *R. semicircularis hosidei*, *R. spina* and *R. tianmu*) (Fig. 8L–V).

Type 4: Pincer-like structure. The prefemur and prefemoral process are nearly equal in length and together form a closed, ring-like structure. In some species, the prefemoral process is S-shaped (4 species, including *R. chelifera*, *R. davidiani*, *R. diacantha*, and *R. semicircularis semicircularis*) (Fig. 8W–Z).

Type 5: Non-pincer structure. The prefemoral process is reduced or entirely absent. The acropodite is curved at a distinct angle at its tip or specialized into a complex, bifurcated structure (8 species, including *R. contigua*, *R. cucfuongensis*, *R. kabaki*, *R. korolevi*, *R. ochracea*, *R. scutata*, *R. uraensis* and *R. langyaensis* sp. nov.; note that *R. ochracea* is described only in a detailed textual description and lacks gonopod illustrations) (Fig. 8AA–AG). Using this morphological framework, we developed a comprehensive dichotomous key for all known Chinese species of *Riukiaria*. The complete key is attached at the end of the paper.

**Figure 9.**
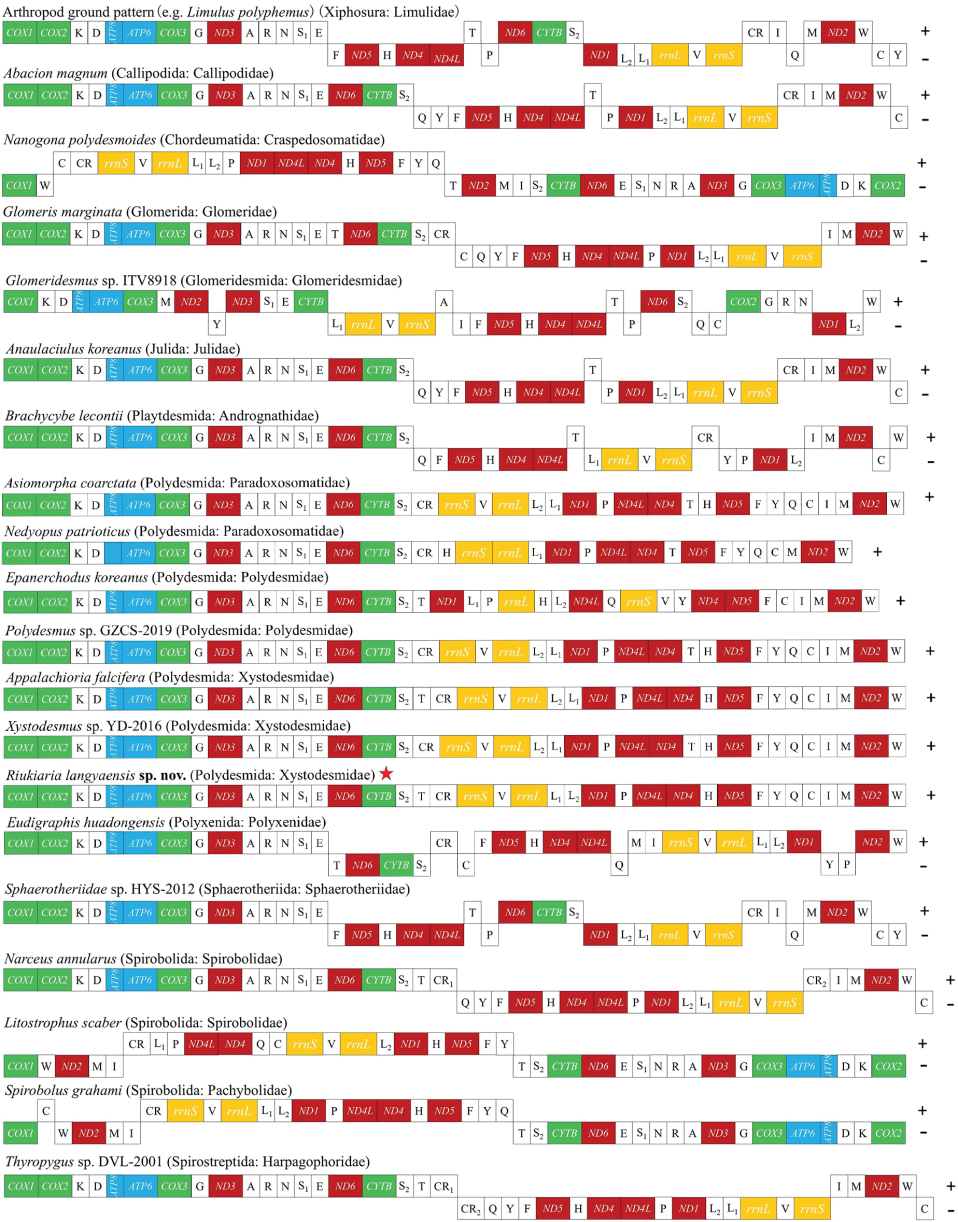
Comparison of mitochondrial gene arrangements among 19 millipede species (including *Riukiaria langyaensis* sp. nov., indicated by a red asterisk). For ease of comparison, the mitogenomes have been linearized and are presented with *COX1* as the starting gene where possible. Different genes are represented by distinct colors. Genes shown above the diagram are encoded on the Heavy strand, while those below are on the Light strand.

Given their rapid evolutionary rates, high information density, and relatively low homoplasy in gene arrangement, complete mitochondrial genomes serve as effective tools for investigating deep-level phylogenetic relationships. In this study, we mapped and compared the mitochondrial gene arrangement patterns of the arthropod ancestor *Limulus polyphemus* and 19 myriapod species (Fig. 9). The results reveal high diversity in mitochondrial gene order among myriapods, with widespread rearrangements. Notably, all mitochondrial genes in the order Polydesmida are located on the same strand. Within the family Xystodesmidae, the three currently sequenced genera (*Appalachioria*, *Xystodesmus*, and *Riukiaria*) each exhibit distinct gene arrangement patterns, suggesting that this feature may hold potential for distinguishing genera. However, due to the limited data currently available, the general applicability of gene arrangement patterns for clarifying intergeneric relationships and even species-level taxonomy within this family requires further evaluation through sequencing additional species. An intriguing scientific question remains whether the significant morphological divergence in gonopod structure within *Riukiaria* (e.g., the non-forceps-shaped Type 5) is associated with its unique mitochondrial gene arrangement.

The taxonomic status of several species within Type 5 has been controversial. Nguyen (2016) erected the new genus *Parariukiaria* to accommodate several species, but Golovatch (2023) recently re-synonymized it with *Riukiaria*. As research progresses, an increasing number of species lacking typical forceps-shaped gonopods are being discovered. Whether such morphologically distinct groups should be recognized as a separate genus is a taxonomically significant issue, the resolution of which awaits more comprehensive, integrated morphological and molecular analyses.

With the accumulation of additional mitogenomic data for this genus and its close relatives, combined with supplementary specimen collection and re-evaluation of morphological characters, the currently unresolved phylogenetic relationships and taxonomic uncertainties are expected to be increasingly reliably resolved.

### Key to species of *Riukiaria* from China, based on males

**Table:** 

1	Gonopods not conforming to the classic pincer-like structure	**2**
–	Gonopods conforming to the classic pincer-like structure (with distinct prefemur, prefemoral process, and acropodite)	**7**
2	Gonopods short, stout, arched and hairy; each ending in two irregularly sinuate, dissimilar, recurved, slender processes	***Riukiaria ochracea***
–	Gonopod structure not as above	**3**
3	Prefemoral process totally missing	**4**
–	Prefemoral process present, but significantly reduced	**5**
4	Gonopod coxae devoid of setae; solenomere very slender, slightly helicoid, acuminate towards tip, very poorly expanded and more clearly curved only subterminally (Fig. 8AD)	***Riukiaria korolevi***
–	Gonopod coxa short, stout, subcylindrical, with two coxal macrosectae; telopodite and prefemur with dense setae on left side; acropodite inflated, oval, distinctly curved; solenomere apical, bifurcated (Fig. 8AG)	***Riukiaria langyaensis* sp. nov**.
5	Prefemoral process a rather strong, stout, suberect, spiniform tooth, very much shorter than the acropodite; distal ¼ of solenomere bifurcate, both branches curved, flagelliform and acuminate towards tip (Fig. 8AC)	***Riukiaria kabaki***
–	Prefemoral process not a spiniform tooth	**6**
6	Prefemur with a rather slender prefemoral process, which is contiguous with the femur; telopodites bristled and branched on the ending (Fig. 8AA)	***Riukiaria contigua***
–	Prefemur with long hairs and a small prefemoral process, which is branched; telopodites slightly broaden, not bidentate, the tip curved (Fig. 8AE)	***Riukiaria uraensis***
7	Prefemur and prefemoral process nearly equal in length and together forming a closed, ring-like structure (Fig. 8X)	***Riukiaria davidiani***
–	Prefemur and prefemoral process not forming a closed loop	**8**
8	Both prefemur and acropodite bent at approximately a 90° angle	**9**
–	Prefemur and acropodite not bent at a 90° angle	**11**
9	Prefemoral process relatively complex, nearly as long as the acropodite; distal half of prefemoral process biramous, tip bifid; tip of solenomere subunciform and subacuminate (Fig. 8J)	***Riukiaria martensi***
–	Prefemoral process simple, not complexly branched	**10**
10	Prefemoral process a strong, stout, slightly curved spine, very much shorter than the acropodite; distal half of solenomere clearly constricted, irregularly attenuating, sigmoid, curved and acuminate towards tip (Fig. 8I)	***Riukiaria belousovi***
–	Prefemoral process spatulate, less than half as long as a slender and progressively attenuating solenomere, distally subgeniculate, tip acuminate (Fig. 8K)	***Riukiaria spatuliformis***
11	Prefemur and prefemoral process compactly arranged; prefemoral process slightly shorter than prefemur and not forming a closed loop with it	**12**
–	Prefemur and prefemoral process not compactly arranged, or with complex morphology	**15**
12	Prefemoral process extremely slender, spine-like bending mesially, as long as the long, scythe-shaped acropodite (Fig. 8U)	***Riukiaria spina***
–	Prefemoral process not an extremely slender, spine-like process	**13**
13	Prefemoral process arcuate, somewhat curved laterally (Fig. 8M)	***Riukiaria chinensis***
–	Prefemoral process not arcuate	**14**
14	Prefemoral process spoon-shaped, thin and enlarged in apical ^1^/_3_ (Fig. 8V)	***Riukiaria tianmu***
–	Prefemoral process long, curved inward in the anterior two-thirds with a distinct lateral ridge, the posterior one-third abruptly slenderized and curved arcwise towards the acropodite (Fig. 8L)	***Riukiaria capaca***
15	Both prefemur and prefemoral process distally trifurcate (Fig. 8D)	***Riukiaria holstii***
–	Prefemur and prefemoral process not distally trifurcate	**16**
16	Telopodite long, broad, the distal quarter bent at a right angle, like a curved band; prefemoral process broad, pointed, straight, much shorter than the telopodite (Fig. 8C)	***Riukiaria geniculata***
–	Telopodite structure not a distally right-angled, band-like form	**17**
17	Acropodite broad, long, curved, near the end, a small, thorn-like tip branches off laterally (Fig. 8F)	***Riukiaria spiralipes***
–	Acropodite without a small, lateral, thorn-like tip near the end	**18**
18	Acropodite moderately slender, somewhat curved, the narrow end strongly and suddenly twice twisted helically; prefemoral process very large, broad, long-triangular, suddenly becoming slender and sharper at the end (Fig. 8G)	***Riukiaria taiwana***
–	Prefemur with a large prefemoral process almost the same size and cohesive in the same position; telopodites curved and with a small branch (Fig. 8B)	***Riukiaria cohaesiva***

## Supplementary Material

XML Treatment for Riukiaria

XML Treatment for Riukiaria langyaensis

## Additional information

### Use of AI

No use of AI was reported.

### Author ORCIDs

Zewu Han https://orcid.org/0009-0001-8254-2051

Xin Liu https://orcid.org/0009-0000-3486-8666

Jiajia Wang https://orcid.org/0000-0002-1843-3977

Yan Dong https://orcid.org/0000-0001-6562-2511

### Data availability

All data supporting the findings of this study are available in the main text or the National Center for Biotechnology Information (NCBI) GenBank database (www.ncbi.nlm.nih.gov/Genbank) under accession number PX436095.

## Supplementary materials

Supplementary material 1 Mitochondrial genome analysis-related charts Data type docx Explanation note It mainly includes the analysis of mitochondrial genome structure, relative synonymous codon usage (RSCU) and codon usage bias, as well as species information of Diplopoda based on the comparison of mitochondrial gene arrangement patterns. https://binary.pensoft.net/file/1558606This dataset is made available under the Open Database License (http://opendatacommons.org/licenses/odbl/1.0/). The Open Database License (ODbL) is a license agreement intended to allow users to freely share, modify, and use this Dataset while maintaining this same freedom for others, provided that the original source and author(s) are credited. Zewu Han, Xin Liu, Jiajia Wang, Yan Dong

Supplementary material 2 Phylogenetic analysis Data type docx Explanation note It primarily includes the methodology and results of the phylogenetic analysis, as well as the phylogenetic tree. https://binary.pensoft.net/file/1558607This dataset is made available under the Open Database License (http://opendatacommons.org/licenses/odbl/1.0/). The Open Database License (ODbL) is a license agreement intended to allow users to freely share, modify, and use this Dataset while maintaining this same freedom for others, provided that the original source and author(s) are credited. Zewu Han, Xin Liu, Jiajia Wang, Yan Dong

Supplementary material 3 Phylogenetic tree Data type pdf Explanation note The vector file of the phylogenetic tree. https://binary.pensoft.net/file/1558608This dataset is made available under the Open Database License (http://opendatacommons.org/licenses/odbl/1.0/). The Open Database License (ODbL) is a license agreement intended to allow users to freely share, modify, and use this Dataset while maintaining this same freedom for others, provided that the original source and author(s) are credited. Zewu Han, Xin Liu, Jiajia Wang, Yan Dong

Supplementary material 4 Phylogenetic tree Data type nex Explanation note The NEXUS format file of the phylogenetic tree. https://binary.pensoft.net/file/1558609This dataset is made available under the Open Database License (http://opendatacommons.org/licenses/odbl/1.0/). The Open Database License (ODbL) is a license agreement intended to allow users to freely share, modify, and use this Dataset while maintaining this same freedom for others, provided that the original source and author(s) are credited. Zewu Han, Xin Liu, Jiajia Wang, Yan Dong
