# Supplementary material for: Description of a new species of the genus Riukiaria (Diplopoda, Polydesmida, Xystodesmidae) from eastern China, with the characterization of its complete mitochondrial genome
Source: Zookeys. 2026 Mar 10;1272:315–35. doi: 10.3897/zookeys.1272.182977 (PMC12997040; doi:10.3897/zookeys.1272.182977)
Supplement: Supplementary material 1 — Mitochondrial genome analysis-related charts [file zookeys-1272-315_article-182977__-s001.docx]

**Supplementary table S1.** Features of the mitochondrial genome of *Riukiaria langyaensis* sp. nov. The direction of coding strands is indicated by major strand (+).

| Gene | Location | | Length (bp) | Anticodon | Intergenic | Codon | | Strand |
| --- | --- | --- | --- | --- | --- | --- | --- | --- |
|  | From | To |  |  |  | Start | Stop |  |
| *COX1* | 1 | 1533 | 1533 |  | +3 | ATG | TAA | + |
| *COX2* | 1537 | 2213 | 677 |  | 0 | ATG | TA | + |
| *trnK* | 2214 | 2277 | 64 | CTT | 0 |  |  | + |
| *trnD* | 2277 | 2339 | 63 | GTC | 0 |  |  | + |
| *ATP8* | 2340 | 2495 | 156 |  | -1 | ATG | TAG | + |
| *ATP6* | 2495 | 3193 | 682 |  | -38 | ATG | TAG | + |
| *COX3* | 3156 | 3941 | 786 |  | +2 | ATG | TAA | + |
| *trnG* | 3944 | 4006 | 63 | TCC | 0 |  |  | + |
| *ND3* | 4007 | 4360 | 354 |  | -2 | ATG | TAG | + |
| *trnA* | 4359 | 4418 | 60 | TGC | 0 |  |  | + |
| *trnR* | 4419 | 4479 | 61 | TCG | -3 |  |  | + |
| *trnN* | 4477 | 4537 | 61 | GTT | 0 |  |  | + |
| *trnS_1_* | 4538 | 4595 | 58 | GCT | 0 |  |  | + |
| *trnE* | 4596 | 4657 | 62 | TTC | +3 |  |  | + |
| *ND6* | 4661 | 5140 | 480 |  | -14 | ATT | TAA | + |
| *CYTB* | 5127 | 6240 | 1114 |  | 0 | ATG | T | + |
| *TrnS_2_* | 6241 | 6297 | 57 | TGA | 0 |  |  | + |
| *trnT* | 6298 | 6358 | 61 | TGT | 0 |  |  | + |
| *CR* | 6359 | 6962 | 604 |  | 0 |  |  | / |
| *rrnS* | 6963 | 7704 | 742 |  | +4 |  |  | + |
| *trnV* | 7709 | 7772 | 64 | TAC | 0 |  |  | + |
| *rrnL* | 7773 | 9048 | 1276 |  | -40 |  |  | + |
| *trnL_1_* | 9009 | 9066 | 58 | TAG | 0 |  |  | + |
| *trnL_2_* | 9067 | 9127 | 61 | TAA | -3 |  |  | + |
| *ND1* | 9125 | 10052 | 928 |  | 0 | ATA | T | + |
| *trnP* | 10053 | 10117 | 65 | TGG | +7 |  |  | + |
| *ND4L* | 10125 | 10406 | 282 | TAG | -7 | ATG | TAG | + |
| *ND4* | 10400 | 11740 | 1341 |  | +1 | ATG | TAG | + |
| *trnH* | 11742 | 11806 | 65 | GTG | +2 |  |  | + |
| *ND5* | 11809 | 13507 | 1699 |  | -0 | ATG | T | + |
| *trnF* | 13508 | 13571 | 64 | GAA | 0 |  |  | + |
| *trnY* | 13572 | 13633 | 62 | GTA | 0 |  |  | + |
| *trnQ* | 13634 | 13698 | 65 | TTG | -1 |  |  | + |
| *trnC* | 13698 | 13763 | 66 | GCA | -4 |  |  | + |
| *trnI* | 13760 | 13820 | 61 | GAT | 0 |  |  | + |
| *trnM* | 13821 | 19844 | 64 | CAT | 0 |  |  | + |
| *ND2* | 13885 | 14895 | 1011 |  | -2 | GTG | TAA | + |
| *trnW* | 14894 | 14954 | 60 | TCA | +1 |  |  | + |

**Supplementary table S2.** Species classification information and GenBank accession numbers for analysis.

| Order | Famliy | Species | Length(bp) | Accession |
| --- | --- | --- | --- | --- |
| Callipodida | Callipodidae | *Abacion magnum* | 15160 | NC_021932 |
| Chordeumatida | Craspedosomatidae | *Nanogona polydesmoides* | 16547 | OZ231999 |
| Glomerida | Glomeridae | *Glomeris marginata* | 16541 | MT881677 |
| Glomeridesmida | Glomeridesmidae | *Glomeridesmus* sp. ITV-8918 | 14848 | MG905160 |
| Julida | Julidae | *Anaulaciulus koreanus* | 14916 | NC_034656 |
| Playtdesmida | Andrognathidae | *Brachycybe lecontii* | 15115 | NC_021934 |
| Polydesmida | Paradoxosomatidae | *Asiomorpha coarctata* | 15644 | KU721885 |
|  |  | *Nedyopus patrioticus* | 15814 | OR755973 |
|  | Polydesmidae | *Epanerchodus koreanus* | 15581 | NC_051495 |
|  |  | *Polydesmus* sp. GZCS-2019 | 15036 | MZ677220 |
|  | Xystodesmidae | *Appalachioria falcifera* | 15282 | NC_021933 |
|  |  | *Xystodesmus* sp. YD-2016 | 15791 | KU721886 |
|  |  | *Riukiaria langyaensis* **sp. nov.** | 14954 | PX436095 |
| Polyxenida | Polyxenidae | *Eudigraphis huadongensis* | 15206 | PV243313 |
| Sphaerotheriida | Sphaerotheriidae | *Sphaerotheriidae* sp. HYS-2012 | 14970 | NC_018361 |
| Spirobolida | Spirobolidae | *Narceus annularis* | 14868 | NC_003343 |
|  |  | *Spirobolus grahami* | 14875 | NC_082185 |
|  | Pachybolidae | *Litostrophus scaber* | 15081 | NC_081977 |
| Spirostreptida | Harpagophoridae | *Thyropygus* sp. DVL-2001 | 15133 | NC_003344 |


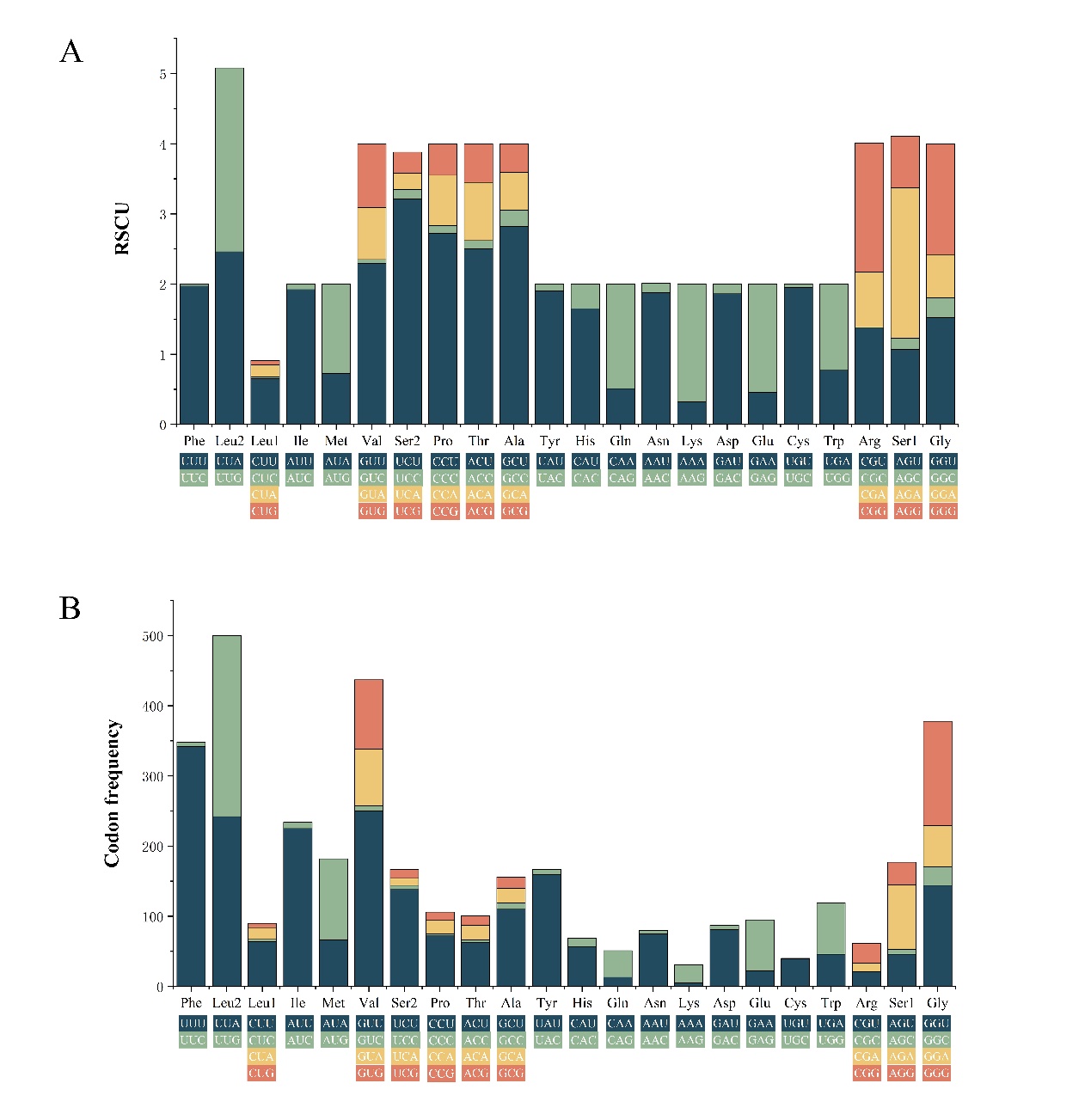
 **Supplementary figure S1.** **A** relative Synonymous Codon Usage (RSCU) in the mitochondrial genome of *Riukiaria langyaensis* sp. nov. **B** Codon usage frequency in the mitochondrial genome of *Riukiaria langyaensis* sp. nov.. The codon families are arranged in alphabetical order along the horizontal axis.
