# Supplementary material for: Description of a new species of the genus Riukiaria (Diplopoda, Polydesmida, Xystodesmidae) from eastern China, with the characterization of its complete mitochondrial genome
Source: Zookeys. 2026 Mar 10;1272:315–35. doi: 10.3897/zookeys.1272.182977 (PMC12997040; doi:10.3897/zookeys.1272.182977)
Supplement: Supplementary material 2 — Phylogenetic analysis [file zookeys-1272-315_article-182977__-s002.docx]

**Supplementary Text S1：**

**Materials and methods**

***Phylogenetic analysis.***

For the phylogenetic analysis, *COX1* sequences from 30 species across 14 genera within the tribe Xystodesmini were obtained from the National Center for Biotechnology Information (NCBI). *Euryurus orestes* and *Euryurus leachii* (tribe Euryurini) were designated as the outgroup (see Appendix for species details).

Phylogenetic reconstruction was performed using Bayesian inference implemented in PhyloSuite v2. Sequences were aligned with MAFFT v7.505 (Katoh & Standley, 2013) using the “E‑INS‑i (accurate)” strategy under standard alignment mode. Gap regions were trimmed with trimAI v1.2rev57 (Capella‑Gutiérrez et al., 2009) under the “‑automated1” setting. The best‑fit partitioning scheme and substitution model were selected from a single predefined partition using PartitionFinder2 v2.1.1 (Lanfear et al., 2016) under the greedy search algorithm and the AICc criterion. Finally, a partitioned Bayesian analysis was conducted in MrBayes v3.2.7a (Ronquist et al., 2012) with two independent runs of 2 million generations each; the first 25% of sampled trees were discarded as burn‑in.

**Phylogenetic results**

The Bayesian phylogeny inferred from the mitochondrial *COX1* gene reveals poorly resolved boundaries among the genus *Riukiaria* and its related genera *Xystodesmus*, *Levizonus*, and *Yaetakaria*. Species from these genera are extensively intermingled across the tree, failing to form distinct monophyletic clades and instead showing para- or polyphyletic patterns.

**
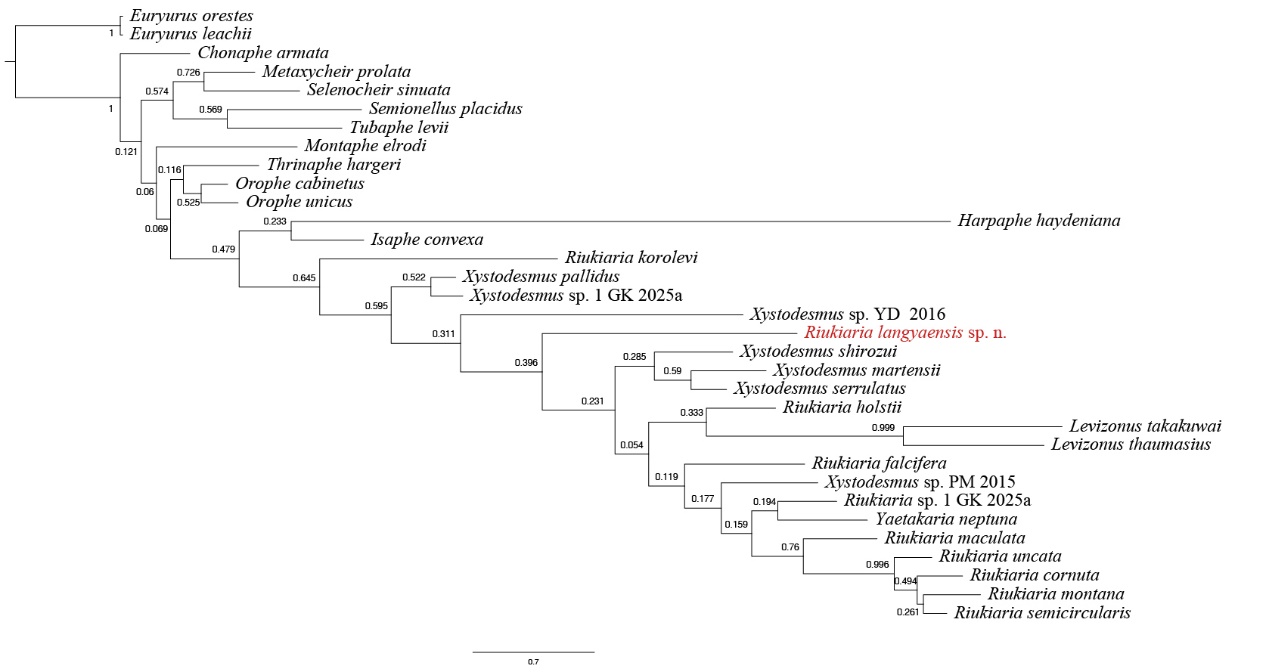
**

**Supplementary figure S2.** Rooted phylogenetic tree based on cytochrome c oxidase subunit I (*COX1*) sequence data. The Bayesian posterior probability (BPP) is shown at each node. Species sequenced in this study are highlighted in red.

**References**

Capella-Gutierrez S, Silla-Martinez JM, Gabaldón T (2009) trimAl: a tool for automated alignment trimming in large-scale phylogenetic analyses. Bioinformatics 25(15): 1972–1973. <https://doi.org/10.1093/bioinformatics/btp348>

Katoh K, Standley DM (2013) MAFFT multiple sequence alignment software version 7: improvements in performance and usability. Molecular Biology and Evolution 30(4): 772–780. <https://doi.org/10.1093/molbev/mst010>

Lanfear R, Frandsen PB, Wright AM, Senfeld T, Calcott B (2016) PartitionFinder 2: new methods for selecting partitioned models of evolution for molecular and morphological phylogenetic analyses. Molecular Biology and Evolution 34(3): 772–773. <https://doi.org/10.1093/molbev/msw260>

Ronquist F, Teslenko M, van der Mark P, Ayres DL, Darling A, Höhna S, Larget B, Liu L, Suchard MA, Huelsenbeck JP (2012) MrBayes 3.2: efficient Bayesian phylogenetic inference and model choice across a large model space. Systematic Biology 61(3): 539–542. <https://doi.org/10.1093/sysbio/sys029>

Zhao D, Ye T, Gao F, Jakovlić I, La Q, Tong Y, Liu X, Song R, Liu F, Lian Z, Zou H, Li W, Wang G, Zeng B, Zhang D (2025) PhyloSuite v2: The Development of an All‐in‐One, Efficient and Visualization‐Oriented Suite for Molecular Dating Analysis and Other Advanced Features. iMeta e70095. <https://doi.org/10.1002/imt2.70095>
