# Supplementary figures and images for: Description of a new species of the genus Riukiaria (Diplopoda, Polydesmida, Xystodesmidae) from eastern China, with the characterization of its complete mitochondrial genome
Source: Zookeys. 2026 Mar 10;1272:315–35. doi: 10.3897/zookeys.1272.182977 (PMC12997040; doi:10.3897/zookeys.1272.182977)

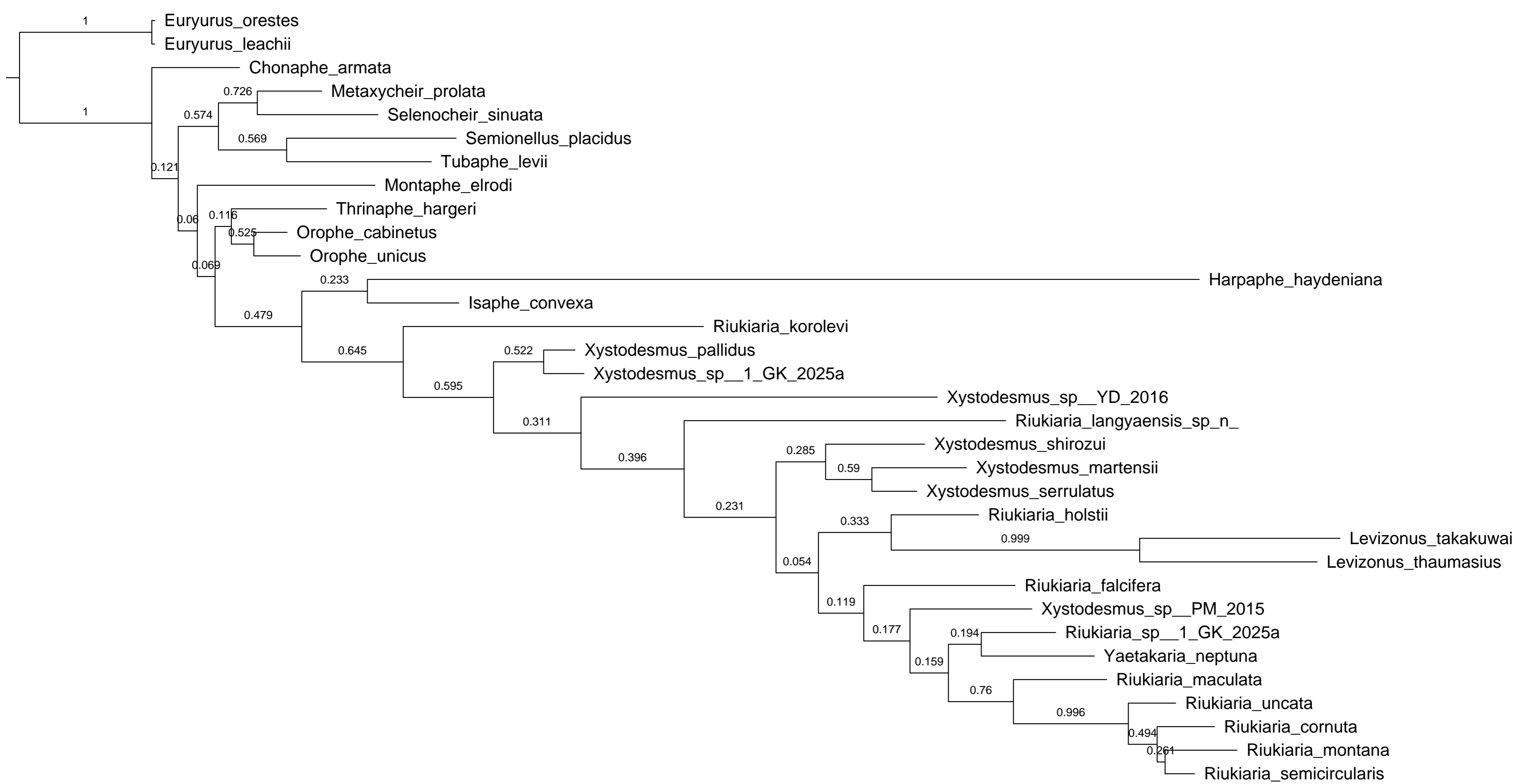

0.7

Supplement: Supplementary material 3 — Phylogenetic tree [file zookeys-1272-315_article-182977__-s003.pdf]
